# Reducing refusals of care through improved personal care interactions between caregivers and people with dementia: protocol for a realist synthesis

**DOI:** 10.1136/bmjopen-2024-088149

**Published:** 2024-08-28

**Authors:** Tamara Backhouse, Anne Killett, Reed WR Bratches, Eneida Mioshi

**Affiliations:** 1 School of Health Sciences, University of East Anglia, Norwich, UK; 2 School of Nursing - Nursing Acute, Chronic & Continuing Care, The University of Alabama at Birmingham, Birmingham, Alabama, USA

**Keywords:** Dementia, Caregivers, Review, Nursing Care

## Abstract

**Abstract:**

**Introduction:**

People with dementia develop progressive difficulties conducting basic activities of daily living, often requiring considerable assistance from caregivers. Many people with dementia, particularly in the advanced stages, can refuse assistance with care leading to difficult interactions. The ways in which refusals of care can be best reduced are unknown. Using a realist approach, this study aims to develop and refine evidence-based programme theories showing which mechanisms of interventions contribute to reducing refusals of care between caregivers and people with dementia, in which contexts, how and why.

**Methods and analysis:**

The realist synthesis will be conducted in three iterative stages.

Stage 1 will develop initial programme theories through secondary analysis of caregivers and persons with dementia interviews and observations, a preliminary exploratory literature review and team discussions. After initial programme theory development, the focus of the synthesis will be decided by the study team.

Stage 2 will involve conducting focused, iterative and targeted literature searches to test and refine our initial programme theories considering the evidence for each setting: hospital, care home, home care and family. Data synthesis will use a realist lens to examine what works for whom in what circumstances and how, and organise related evidence to context-mechanism-outcome configurations whenever possible.

Stage 3 will use stakeholder interviews to explore reactions to the programme theories and enhance validity after integration of these findings, recommendations and conclusions will be developed.

**Ethics and dissemination:**

The NHS Social Care Research Ethics Committee has approved the interview stage of this study (REC reference: 24/IEC08/0007; IRAS project ID: 338274). Informed consent will be obtained from all interviewees prior to data collection. Findings will be disseminated via peer-reviewed publications, conference presentations and accessible information for key stakeholders.

**PROPSPERO registration number:**

CRD42024496072.

**STRENGTHS AND LIMITATIONS OF THIS STUDY**

- A realist approach will enable the complexity of refusal-of-care interactions to be considered, examining what works for whom, how and under what circumstances.
- A strength of this study is the diverse range of sources used to identify causal insights about mechanisms underlying refusals of care.
- Potentially relevant insights written in different languages may be missed due to this study only including those written in English.
- The project team may have to prioritise key features of care interactions or settings based on relevance, plausibility and preliminary evidence levels.

## Introduction

 It is estimated that there are 900 000 people living with dementia in the UK, a number projected to increase to 1.6 million by 2040.^1^ Dementia is a progressive disease and people living with it can develop multiple symptoms such as difficulties with cognition, memory, communication, mobility and understanding, and mood and behavioural symptoms (eg, apathy and agitation).^2^ As time goes on, these symptoms lead to difficulties in conducting basic activities of daily living (ADLs) such as washing, dressing and going to the toilet.^3^ Consequently, physical support from caregivers becomes essential.^4^

Many people with dementia refuse assistance with care, particularly in the advanced stages.^5^ For example, they can verbally refuse, stiffen their body or push the caregiver away.^6^ Refusals of care can be caused by the caregiver approach such as not listening to the person, pressing on with care when the person is uncertain^7^ or using negative or patronising communication.^8 9^ Other causes include the person not understanding caregiver intentions,^10 11^ having unmet needs such as being in pain or hungry^5 9^ or experiencing psychotic symptoms.^11 12^

Refusals of care are often difficult for caregivers to manage and can cause distress to both caregivers and the person with dementia. If care is not provided the person with dementia can experience poor hygiene, soreness, infections, neglect and other issues such as threats to their dignity due to becoming unkempt or malodourous.^13^ Caregivers often feel pressure to complete care activities, and this can lead to them seeking prescriptions for psychotropic medications, employing controlled restraint or drawing on specialist support services such as mental health or dementia intensive support teams.^13^,^16^ Refusals of care are relational and occur within interactions,^10^ therefore, improving personal care interactions may reduce refusals of care. However, what may work, how, for whom and under what circumstances is unknown.

Formal dementia care can often be task driven^17^ focusing on assisting people with basic ADLs quickly so staff can move on to the next planned action due to time constraints. Often paid carers must contend with staff shortages,^18^ high turnover^19^ and limited training opportunities.^20^ Family carers may have more time but have other stressors such as coping with the 24-hour nature of caring, social isolation, and a need for training and knowledge.^21 22^ The National Institute for Health and Care Excellence (NICE) guidance^23^ recommends using non-pharmacological interventions rather than medications to manage behaviours which are challenging for caregivers; however, no clearly evidenced alternative intervention has been identified to reduce refusals of care.

A best evidence review assessing interventions in care homes to reduce refusals of care found low-level evidence for a person-centred approach, music interventions and ability focused approaches.^24^ A more recent systematic review^14^ focusing on all care settings found most evidence for playing recorded music during care activities and bathing techniques such as person-centred showering or washing conducted while the person is in bed for reducing refusals of care. Elderspeak (a style of patronising communication, eg, using overly endearing terms and tones, slow speech and overinclusive language) and controlling or negative communication styles were associated with refusals of care.^14^ These reviews provide some indication of what may work, however, exactly which interventions or mechanisms of interventions work for whom, and how, in what contexts is unknown.

Therefore, we will conduct a realist synthesis aiming to identify strategies and mechanisms of interventions between caregivers and people with dementia that contribute to reducing refusals of care and determine how they work in which contexts and why.

We will:

1. Identify how interventions to reduce refusals of personal care for people with dementia are thought to achieve better care interactions.
2. Develop programme theories describing contexts and causal mechanisms of programmes/programme features where caregivers can improve personal care interactions for people with dementia, which results in positive outcomes.
3. Identify and evidence what works, in what circumstances and how.

## Methods and analysis

### Study design

**Figure 1:**
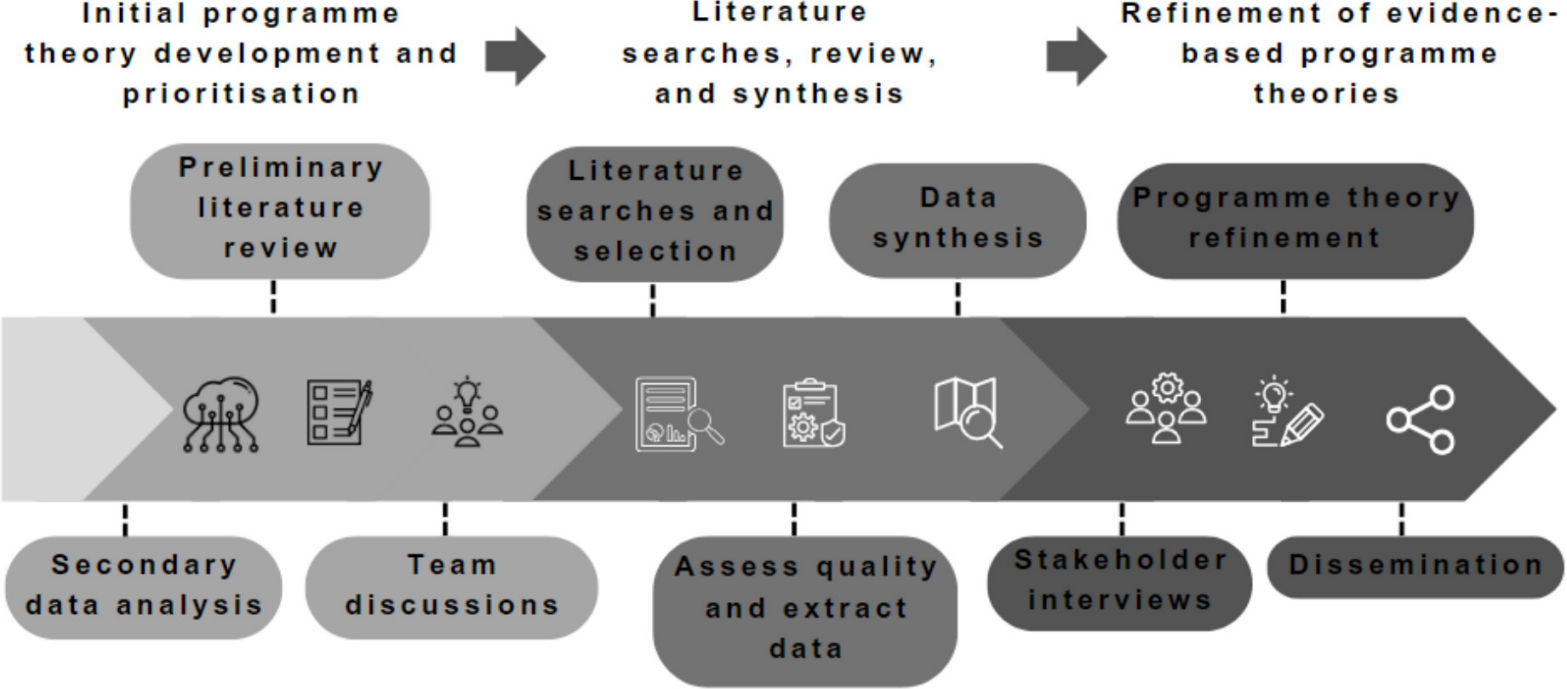
Study design.

Realist synthesis was chosen as appropriate for this review since it can examine complex interventions or intervention components accounting for context, mechanisms and outcomes, providing evidence-based theories and pragmatic conclusions.^25 26^ This realist synthesis will be conducted between November 2023 and January 2025, it draws on Pawson *et al*’s^27^ key steps in realist review and has three stages: (1) initial programme theory development and prioritisation (November 2023–April 2024), (2) literature search, review and synthesis (April 2024–September 2024) and (3) refinement of evidence-based programme theories (September 2024–January 2025) (see figure 1).

### Patient and public involvement

This project has been informed by public involvement from the outset. The idea to work towards improving personal care interactions to reduce refusals of care for people with dementia came from workshops with care-home staff and family carers as part of the Pro-CARE study (the first author’s Alzheimer’s Society funded fellowship). Keeping caregiver and person with dementia perspectives informing our work, we consulted with the Stevenage Dementia Involvement Group (September 2023) about factors important in caregiver/recipient interactions to feed into initial programme theory work. During the review process and dissemination, two public representatives will contribute regularly as advisors, providing their thoughts on initial programme theories, reviewing and shaping evidenced-based theories and synthesising stakeholder evidence and contributing to the development of conclusions and recommendations.

### Stage 1: initial programme theory development and prioritisation

Initial programme theories will be generated in three ways: assessing interview transcripts and video-recorded personal care interactions, preliminary scoping of key literature and team discussions.

#### Assessing video-recorded personal care interactions and interview transcripts

To ensure that caregivers and people with dementia’s experiences about refusals of care and personal care feed into our initial programme theories, we will conduct a secondary data analysis of interviews and observations conducted for the Pro-CARE Study. This study was a fellowship for the first author funded by the Alzheimer’s Society in England. The programme of research examined refusals of care and personal care interactions between caregivers and people with advanced dementia. Interviews were conducted with 20 family carers, 12 care-home staff,^28^ 17 home-care workers^29^ and 13 people with dementia receiving assistance with their personal care. Interviews focused on experiences of personal care assistance and refusals. Observations consisted of 26 video-recorded personal care interactions between 14 family carer or care-home staff/people with dementia dyads and covered activities where the person with dementia was clothed such as teeth cleaning, hair washing and shaving (3 hours of footage in total).^7^

The first author will analyse these datasets with a realist lens examining a range of complexities that potentially interact. The analysis will seek to explain the factors involved in actions for care, reactions to care interactions and potentially helpful strategies for working with refusals of care. Data will be categorised into tentative programme theories and possible underlying causal mechanisms of actions will be developed about how approaches worked and if possible, what needed to be in place to support their use. Comparison within and between participant categories and observation or interview-derived data will enable identification of cross-cutting causal insights. Causal insights will be taken forward and evidence of data retained to enable an audit trail of theoretical development and decision-making. Team discussions will assist in theory building, interrogate initial ideas and add further insight to initial theories.

#### Preliminary review

An initial exploratory review of key literature will take place^27^ aiming to identify the breadth of interventions and intervention components to feed into the initial programme theories with the aim to find cross-cutting mechanisms. Targeted searching will include key articles, for example, those included in a recent systematic review on strategies to reduce or manage refusals of care.^14^ In addition, articles and interventions known by the study team involving caregiver interactions with people with dementia such as namaste care,^30^ The Voice Study dementia communication skills training^31^ and Humanitude Care^32^ will be assessed.

#### Team discussions

The study team has expertise in occupational therapy, personal care assistance, dementia care research, and care home, family carer, and hospital care settings. Using theoretical insights gleaned from the secondary data analysis and preliminary review, we will develop initial IF, THEN, BECAUSE statements. Project team theory building will involve considering insights from data, initial literature and their own expertise to generate causal statements, which can be developed into context-mechanism-outcome configurations at the end of stage 2. From these initial programme theories, the team will agree on the scope and focus of the realist synthesis, if necessary, prioritising initial programme theories to take forward based on their relevance, plausibility and preliminary evidence levels. The team will design a theoretically based framework to be populated with evidence.^27^ Existing mid-range theories will be sought through targeted searches led both from the existing knowledge of the research team and from the developing programme theories. These will be examined and, if relevant, incorporated to guide our theory building.

### Stage 2: literature search, review and synthesis

#### Searches

The search strategies have been developed by the study team (see online supplemental file), aiming to find literature that will generate, evidence and develop programme theories to, if possible, specify context, mechanism and outcome configurations.^33 34^ Further searches will be conducted iteratively to explore individual programme theories as necessary as the synthesis progresses.

The searches have three strands: dementia, refusals of care and personal care/ADLs. We will purposively search through the following databases: MEDLINE, EMBASE, PsycINFO, CINAHL, CENTRAL—Cochrane Central Register of Controlled Trials and the Social Sciences Citation Index. Searches will be adapted depending on the database used. We will search grey literature sources such as practice-focused publications and organisational websites such as the Alzheimer’s Society. The searches will be restricted to the English language and from 2000 onwards to reflect the time when Kitwood’s person-centred care ethos^35 36^ started to influence dementia care. EndNote reference manager will be used to collate citations and identify duplicates, from our searches.

#### Selection

Selection will be carried out by one reviewer with independent screening of a subset of documents by a second reviewer to support quality assurance. Selection will be based on each publication’s relevance and contribution to theory building. Included studies will focus on refusals of personal care or personal care interactions with people living with dementia, be related to any of four care settings (care home, family home, hospital, home care), include an intervention or examine a strategy to reduce refusals of care or improve personal care for people with dementia and be in the English language. Publications not focused on personal care interactions between people with dementia and caregivers or those focused on end-of-life care will be excluded. To maximise potential understanding any type of peer-reviewed study will be eligible to be included in the review. Therefore, no restrictions will be placed on the study design.

We are interested in whether interventions or components of interventions employed to reduce or manage refusals of personal care in dementia are successful or not, how they may work, for whom and under what circumstances. Depending on retrieved literature and time resources, we will use a quota system, initially assessing 10 studies for each setting: hospital, care home, home care and family, prioritising those with the most relevance to theory building. If limited literature is found, we will examine theoretically relatable practices such as interactions with people with dementia for other purposes, and/or personal care interactions in other groups, for example, learning disability or other neurological or psychiatric conditions. Contextual factors may include the value ascribed to personal care, suitability of the environment, espoused ethos of care, time available, caregiver skills, and symptoms and stage of dementia.

#### Quality appraisal

Quality appraisals will focus on assessments of relevance, richness and rigour,^27 37^ not appraising for methodological quality but assessing all parts of papers including the introduction and discussion for causal thinking and insights.^38^ Relevance will be judged on whether or not the resource contains data applicable to the topic area or programme theories, and richness on whether the resource can contribute sufficiently to theory building or testing.^37 39^ Rigour will be judged on the extent to which the method generating the insight is credible and trustworthy^39^ and whether the theory is coherent.^37^ Where there are difficulties interpreting quality or uncertainties surrounding judgements, articles will be discussed between two reviewers, three if no consensus is reached. Depending on whether other evidence is available for that programme theory, resources with low relevance and richness will be excluded. Where concepts such as refusals of care are not portrayed accurately or the meaning authors are ascribing mismatches our conceptualisation (such as those centred on non-adherence or refusal of medical procedures), we will exclude articles. However, articles offering alternative explanations within our conceptualisation will be included and used to challenge biases and team interpretations.

#### Extraction

A bespoke form aimed at populating the theoretically based framework with evidence linking to initial IF/THEN/BECAUSE and context-mechanism-outcome configurations for testing initial programme theories will be developed. Data extracted are likely to include:

- Study information: authors, title, journal, country of origin, publication date.
- Setting and participants: care setting, sample size and composition including stage of dementia and caregiver type.
- Methods: design, analysis
- Context description: contextual factors including refusal information.
- Intervention/interaction description: inputs, components, processes, mechanisms resource, mechanisms reasons/reactions, outcomes.
- Evidence relating to initial programme theories.

The form will be piloted and refined to make sure it supports detailed information about studies, interventions and intervention components. Data will be extracted from included studies and grey literature by one reviewer (TB) with independent screening of a subset of documents and extraction by a second reviewer to support quality assurance. Data extraction will initially be kept separate for each care context.

#### Synthesis

Synthesis will be exploratory, revising the initial programme theories considering the data to develop evidence-based context-mechanism-outcome configurations. The aim will be to evidence theories related to strategies and mechanisms of interventions between caregivers and people with dementia that contribute to reducing refusals of care and determine how they work in which contexts, why and for whom. One researcher (TB) will read extracted data and relevant texts again, focusing on the theoretical underpinnings of the programme theories considering meanings, theories and rival theories, making notes, establishing connections between concepts. Consideration will be given to both intended and unintended causal insights. Prioritised initial programme theories will be populated with relevant extracted data generally and if possible, under context-mechanism-outcome configurations, including mechanism resources from the intervention and how this changes the reasoning of actors.^40^ Synthesis will occur through ongoing examination of each of the theories and relevant data considering causal insights. Team meetings will examine and interrogate developing theories and context-mechanism-outcome configurations alongside the evidence. If specific evidence is available and only relevant for similar types of interventions or strategies and/or care settings synthesis will occur for subcategories. For example, evidence relevant only to certain settings such as hospitals will be kept separate in our synthesis from programme theories relevant for all settings.

### Stage 3: refinement of evidence-based programme theories

We will use stakeholder interviews to explore reactions to the programme theories and enhance validity.^27^ Stakeholders (up to 15) will be purposively sampled, aiming to provide a range of perspectives and likely to include care-home staff, family carers, home-care workers, care recipients and support team staff such as crisis teams or dementia intensive support staff. Interviews will be conducted iteratively assessing new insights and then conducting more to fill in any gaps where programme theories have not been properly addressed.

Interviews will be semistructured and focus on testing programme theories through participants’ subjective experiences and insights.^41^ Topic guides will be structured around programme theories and may be more explicit or implicit for different stakeholder groups as appropriate. Interviews will adopt a teacher-learning function,^42^ providing enough knowledge of the developed programme theories to participants for them to provide their own subjective theories. The ‘I’ll show-you-my-theory-if-you'll-show-me-yours’(Pawson^42^, p307) process allows researchers to introduce their theories, so participants can contribute to falsify, confirm or refine programme theories. Interviews may be face to face, online or via telephone, they will be audio recorded and transcribed verbatim. Key insights and nuances arising during interviews will be integrated with existing evidence to refine programme theories from which, recommendations and conclusions will be developed.

This synthesis will develop evidence-based and refined programme theories to explain how we may reduce refusals of care between caregivers and people with dementia within the bounds of personal care interactions.^43^ Programme theories will, if possible, each be in context, mechanism, outcome configurations, explaining generative causation. Context refers to aspects such as social norms and interrelationships which trigger causal mechanisms; mechanisms are the hidden psychosocial underlying reactions and/or reasonings of relevant programme participants, and outcomes refer to the conditions influenced by the causal mechanisms.^33 34^

## Ethics and dissemination

Ethical approval for the interviews was obtained from the NHS Social Care Research Ethics Committee has approved this study (REC reference: 24/IEC08/0007; IRAS project ID: 338274). Informed consent will be obtained from all participants prior to data collection. The review will be written up using the quality standards for realist synthesis (RAMESES 1).^44^ Findings will be disseminated via peer-reviewed publications and conference presentations. Additionally, accessible information for key stakeholders such as care home communities, home-care workers and family carers will be disseminated through leaflets and social media.

## supplementary material

10.1136/bmjopen-2024-088149 online supplemental file 1
