# Supplementary material for: Reducing refusals of care through improved personal care interactions between caregivers and people with dementia: protocol for a realist synthesis
Source: BMJ Open. 2024 Aug 28;14(8):e088149. doi: 10.1136/bmjopen-2024-088149 (PMC11367303; doi:10.1136/bmjopen-2024-088149)
Supplement: online supplemental file 1 [file bmjopen-14-8-s001.pdf]

Supplementary file

MAIN SEARCH

Dementia	“Dementia” “Alzheimer*” “Cognitive decline” “Cognitive impair*” “Lewy bod*”
Personal care	“personal care” “ADLs” “activities of daily living” “bath*” “shower*” “wash*” “toilet*” “dress*” “morning routine” “shav*” “eat*” “medication*” “drink*” “nail*” “hair” “care” “teeth” “oral hygiene” “skin care” “feed*”
Refusals	“resistan*” “refus*” “reject*” “non-complian*” “noncomplian*” “complian*” “uncooperative" “obstreperous” “accept*”

(("Dementia" or "Alzheimer*" or "Cognitive decline" or "Cognitive impair*" or "Lewy bod*") and ("personal care" or "ADLs" or "activities of daily living" or "bath*" or "shower*" or "wash*" or "toilet*" or "dress*" or "morning routine" or "shav*" or "eat*" or "medication*" or "drink*" or "nail*" or "hair" or "care" or "teeth" or "oral hygiene" or "skin care" or "feed*") and ("resistan*" or "refus*" or "reject*" or "non-complian*" or "noncomplian*" or "complian*" or "uncooperative" or "obstreperous" or "accept*"))

DATABASES:

OVID MEDLINE

OVID EMBASE

EBSCO PsycINFO

EBSCO CINAHL Ultimate

CENTRAL - Cochrane Central Register of Controlled Trials

Web of Science Social Sciences Citation Index™.

LIMITS:

Year 2000 onwards

English language

Human
