# Supplementary material for: Reducing refusals of care through improved personal care interactions between caregivers and people with dementia: protocol for a realist synthesis
Source: BMJ Open. 2024 Aug 28;14(8):e088149. doi: 10.1136/bmjopen-2024-088149 (PMC11367303; doi:10.1136/bmjopen-2024-088149)
Supplement: Supplementary file 2 [file bmjopen-2024-088149.reviewer_comments.pdf]

PEER REVIEW HISTORY

BMJ Open publishes all reviews undertaken for accepted manuscripts. Reviewers are asked to complete a checklist review form and are provided with free text boxes to elaborate on their assessment. These free text comments are reproduced below.

ARTICLE DETAILS

**TITLE (PROVISIONAL)**	Reducing refusals of care through improved personal care interactions between caregivers and people with dementia: protocol for a realist synthesis
**AUTHORS**	Backhouse, Tamara; Killett, Anne; Bratches, Reed; Mioshi, E

VERSION 1 - REVIEW

**REVIEWER NAME**	***Huijsman, Robbert***
**REVIEWER AFFILIATION**	Erasmus University Rotterdam, Health Policy and Management
**REVIEWER CONFLICT OF INTEREST**	no competing interests
**DATE REVIEW RETURNED**	13-May-2024

**GENERAL COMMENTS**	Thanks for your interesting protocol article.<br>It seems publishable to me, but I have some concerns about your methods which require more in-debt reflections and specifications:<br>- Perhaps you can more clearly/detailed describe what will be the final outcome of your work: is it the whole synthesized program theory itself (single or multiple versions?), or only components of a possible program theory, is it perhaps a conceptual model existing of core concepts and their interrelationships? And what do you foresee as essential (theoretical/conceptual) requirements for program theory/ies when synthesis occurs for subgroups (page 11, lines 38-40?). It sounds too vague/open ended to me.<br>(From Google: Program theory modelling uses three components to describe the program: the program activities or inputs, the intended outcomes or outputs, and the mechanisms through which the intended outcomes are achieved (Reynolds, 1998; Rogers, 2000; Rogers et al, 2000; Sedani & Sechrest, 1999).<br>- The way you describe public involvement (page 6/7) is a bit vague to me and sounds more as a starting point in the first stage than from onset throughout the end stage, as public involvement is not explicitly operationalised in stage 3.<br>- You do not explicate how you are going to map to initial programme theories (page 10); does mapping require a certain procedure/method?<br>- in the Synthesis (page 11) you seem to suggest that you will use CMO logics, without any reference to CMO-methods. Realist evaluation is a logic of inquiry that goes further than merely exploring the surface level inputs and outputs of an intervention, by discerning the psychosocial mechanisms (M), that is, the internal reactions and reasonings, that trigger intervention outcomes (O) in specific contexts (C) of implementation (See for instance De Brún, A., McAuliffe, E. Identifying the context, mechanisms and outcomes underlying collective leadership in teams: building a realist programme theory. BMC Health Serv Res 20, 261 (2020). https://doi.org/10.1186/s12913-020-05129-1).

	- In stage 3: are individual stakeholder interviews the most adequate method or would focus groups (with reflective interaction) a better method, also bringing consensus about the program theory?<br>- Please add timelines to your design and stages.<br><br>Some minor points about references:<br>- There are some inconsistencies in the way you use interpunction for the references in de text (et al. with a dot after all; comma between (last) author and year of publication).<br>- You are overenthusiastically self-referencing to your own publications (nine times!), is that really necessary (perhaps you can use other authors and skip some of your own articles).<br>- You use some rather old references for realist review, are there more recent publications than those of Pawson only?

**REVIEWER NAME**	***Handley, Melanie***
**REVIEWER AFFILIATION**	University of Hertfordshire, CRIPACC
**REVIEWER CONFLICT OF INTEREST**	None
**DATE REVIEW RETURNED**	20-May-2024

**GENERAL COMMENTS**	Thank you for the opportunity to review this protocol of a realist synthesis on reducing refusals through improved care interactions during person care interactions between people living with dementia and caregivers. This protocol sets out an important area of research that will likely result in practical recommendations and guidance for professional and informal carers that can improve the quality of care and relationships for those who need support with personal care activities.<br><br>The manuscript is well written. Below are minor comments for the authors to consider:<br><br>Abstract, Introduction, Line 46: "and for whom" could be deleted as this is already mentioned by referring to caregivers and people with dementia.<br><br>Abstract, Methods and analysis, page 3 line 9: "match informing evidence with" could be "organise related evidence to"<br><br>Strengths and limitations of this study, bullet point 4, page 3 line 50-52: Suggest this bullet point is strengthened to demonstrate the positive reason for prioritising features rather than focusing on time constraints. Realist review does not seek to give an exhaustive account of what is occurring but instead attempts to explain the relationships of the key factors. I suggest the authors instead indicate what they are likely to base their decision on.<br><br>Page 5, line 51: as with the abstract, the "and for whom" could be deleted.<br><br>Page 6, line 3: "develop a programme theory", I suggest this is changed to "Develop programme theories" as this is inline with realist review methodology and would be consistent with the use of programme theories throughout the rest of the manuscript.<br><br>Page 7, lines 5 - 11. The first part of this sentence would benefit from rewording.

	Page 7, lines 52 to 58. Not sure I follow this sentence. Is it that analysing this data will be used to inform topic guides for stakeholder interviews or is it that analysis will seek to explain the factors involved in actions for care, reactions to care interactions and potentially helpful strategies for working with refusals of care? It may be that the sentence is too long and breaking it down into smaller components would help the reader.<br><br>Page 8, line 5: it might be helpful to clarify what "methodological categories" refers to.<br><br>Page 8, line 10: should "adding" be add?<br><br>Page 8, line 52: perhaps the addition of "Existing" before mid-range theories would be helpful. Please also include some commentary on how these theories will be searched for (e.g. will it be from existing knowledge of the research team and targeted searches for theories from the developing programme theories?).<br><br>Page 9, lines 3 - 5. I am not sure I understand what the authors mean by "structured and exploratory searches". Perhaps an additional sentence or two here could be used to clarify the meaning.<br><br>Page 9, line 26: just a suggestion, but I'm wondering if "influence" is a better word than "change" in relation to Kitwood's impact on dementia care.<br><br>Page 10, lines 21 - 25: The sentence starting "Where concepts..." could be read that the authors are only seeking evidence that aligns with their current understanding and initial programme theories. Consider rewording as one of the ways realist review addresses challenges of bias is by adjudicating between alternative explanations.<br><br>Page 10 Line 36: refer to IF THEN BECAUSE statements for consistency.<br><br>Page 10 lines 35 - 39: "context-mechanism-outcome configurations and testing.." I think the and should be for.<br><br>Page 10 line 54: "Mapping to" could instead be "Evidence relating to"<br><br>Page 11 line 12: "redeveloping" could instead be "reworking" or "revising" - redeveloping, to me, suggests starting over rather that using the data to further shape and strengthen initial ideas.<br><br>Page 11, line 21: "One" is missing before "Researcher", "the" is missing before "theoretical underpinnings", "of programme considering" one or several words missing here.<br><br>Page 11, Line 25 - 27: The sentence "Consideration will be given to formal and informal/unintended consequence causal insights." needs rewording.<br><br>Page 11, line 27: "Prioritised initial programme theories" there needs to be an earlier discussion of how initial programme theories will be

	prioritised, what will be the justification for how theories are prioritised.<br><br>Page 11 line 46: the authors state that recommendations and conclusions will be tested and refined in stakeholder interviews - does this mean that working recommendations and conclusions will be developed before interviews with stakeholders? To me, this seems like a separate, later piece of work. I would anticipate interviews would explore stakeholder reactions to the programme theories and enhance the validity but as insights continue to be gathered perhaps agreeing recommendations and conclusions following integrating evidence from these interviews is an additional activity?<br><br>Page 13, line 13 "Then" should be "The".<br><br>Suggestion for throughout the manuscript: to change "Evidenced programme theories" to "Evidence-based programme theories". While the theories will draw on a significant amount of evidence they will only ever remain theories.

VERSION 1 – AUTHOR RESPONSE

Reviewer: 1
Prof. Robbert Huijsman, Erasmus University Rotterdam

Comments to the Author:
Thanks for your interesting protocol article.
It seems publishable to me, but I have some concerns about your methods which require more in-debt reflections and specifications:
- Perhaps you can more clearly/detailed describe what will be the final outcome of your work: is it the whole synthesized program theory itself (single or multiple versions?), or only components of a possible program theory, is it perhaps a conceptual model existing of core concepts and their interrelationships? And what do you foresee as essential (theoretical/conceptual) requirements for program theory/ies when synthesis occurs for subgroups (page 11, lines 38-40?). It sounds too vague/open ended to me.
(From Google: Program theory modelling uses three components to describe the program: the program activities or inputs, the intended outcomes or outputs, and the mechanisms through which the intended outcomes are achieved (Reynolds, 1998; Rogers, 2000; Rogers et al, 2000; Sedani & Sechrest, 1999).
RESPONSE: Many thanks for your comment, we have re-examined other realist syntheses and realist literature to double check appropriate outcomes from this method and now draw on Wong 2018 to state our outcome. In terms of this realist synthesis, these will be whole programme theories, however in terms of a programme theory of the kind needed for a logic model of an intervention/programme (the text you have referred to), these are likely to be partial programme theories, as the scope of our realist synthesis is focussed within the care interaction and not on organisational factors or a programme of care to be evaluated. The text now reads:
“This synthesis will develop evidence-based and refined programme theories to explain how we may reduce refusals of care between caregivers and people with dementia within the bounds of personal care interactions (Wong 2018). Programme theories will, if possible, each be in context, mechanism, outcome configurations, explaining generative causation. Context refers to aspects such as social norms and interrelationships which trigger causal mechanisms; mechanisms are the hidden psychosocial underlying reactions and/or reasonings of relevant programme participants, and outcomes refer to the conditions influenced by the causal mechanisms (De Brún and McAuliffe, 2020;

De Weger et al., 2020)."
In terms of creating programme theories for specific subgroups, we have changed the terminology to subcategories to avoid misinterpretation and have further explained:
"If specific evidence is available and only relevant for similar types of interventions or strategies and/or care settings synthesis will occur for subcategories. For example, evidence relevant only to certain settings such as hospitals will be kept separate in our synthesis from programme theories relevant for all settings."

- The way you describe public involvement (page 6/7) is a bit vague to me and sounds more as a starting point in the first stage than from onset throughout the end stage, as public involvement is not explicitly operationalised in stage 3.
RESPONSE: Many thanks for your comment. This section has been augmented to operationalise involvement for the later stages and now reads:
"During the review process and dissemination two public representatives will contribute regularly as advisors, providing their thoughts on initial programme theories, reviewing and shaping evidence-based theories and synthesised stakeholder evidence, and contributing to the development of conclusions and recommendations."

- You do not explicate how you are going to map to initial programme theories (page 10); does mapping require a certain procedure/method?
RESPONSE: Many thanks for your comment, we have removed the term 'mapping'. We used the term to indicate relating evidence sources to individual programme theories during our extraction process but have changed the text to reflect these actions better. The term 'mapping to initial programme theories' has been changed to " Evidence relating to initial programme theories" in the extraction categories.

- in the Synthesis (page 11) you seem to suggest that you will use CMO logics, without any reference to CMO-methods. Realist evaluation is a logic of inquiry that goes further than merely exploring the surface level inputs and outputs of an intervention, by discerning the psychosocial mechanisms (M), that is, the internal reactions and reasonings, that trigger intervention outcomes (O) in specific contexts (C) of implementation (See for instance De Brún, A., McAuliffe, E. Identifying the context, mechanisms and outcomes underlying collective leadership in teams: building a realist programme theory. BMC Health Serv Res 20, 261 (2020). https://doi.org/10.1186/s12913-020-05129-1).
RESPONSE: Many thanks, we have now addressed this aspect, describing CMO configurations and referring to relevant literature:
"Programme theories will, if possible, each be in context, mechanism, outcome configurations, explaining generative causation. Context refers to aspects such as social norms and interrelationships which trigger causal mechanisms; mechanisms are the hidden psychosocial underlying reactions and/or reasonings of relevant programme participants, and outcomes refer to the conditions influenced by the causal mechanisms (De Brún and McAuliffe, 2020; De Weger et al., 2020)."

- In stage 3: are individual stakeholder interviews the most adequate method or would focus groups (with reflective interaction) a better method, also bringing consensus about the program theory?
RESPONSE: This is an interesting point, which we considered before obtaining ethical approval. Focus groups can be valuable for generating new thoughts about a phenomenon and for consensus building. However, due to the diverse range of stakeholders (care-home staff, family carers, home-care workers, care recipients, and support team staff such as crisis teams or dementia intensive support staff) that we want to include and the potential power imbalances and group dynamics when bringing these people together within focus groups we decided individual interviews would be more appropriate, practical, and allow all participants to voice their thoughts in depth. Getting care workers together for focus groups can be practically very difficult.

- Please add timelines to your design and stages.
RESPONSE: Many thanks, these have now been added:
"This realist synthesis will be conducted between November 2023 and January 2025, it draws on Pawson et al.'s (2005) key steps in realist review and has three stages: 1) initial programme theory development and prioritisation (Nov 2023 - Apr 2024), 2) literature search, review, and synthesis (Apr 2024 – Sept 2024), and 3) refinement of programme theories (Sept 2024 - Jan 2025)"

Some minor points about references:
- There are some inconsistencies in the way you use interpunction for the references in de text (et al. with a dot after all; comma between (last) author and year of publication).
RESPONSE: Many thanks, references have been unified (all with et al with a dot and comma; a comma after last author and year of publication).

- You are overenthusiastically self-referencing to your own publications (nine times!), is that really necessary (perhaps you can use other authors and skip some of your own articles).
RESPONSE: Many thanks for highlighting this, we have completed a lot of work in this research area and hence have relevant evidence to draw on. However, considering your comment, we have reduced the references to our own work (now five articles, key to underpinning this research).

- You use some rather old references for realist review, are there more recent publications than those of Pawson only?
RESPONSE: Many thanks for pointing this out, we have now referred to more recent realist synthesis texts as well as Pawson's seminal texts (for example Dada et al 2023; Mukumbang et al 2020; Jagosh 2019; Wong et at 2013; Wong 2018; De Weger et al. 2020).

Reviewer: 2
Dr. Melanie Handley, University of Hertfordshire

Comments to the Author:
Thank you for the opportunity to review this protocol of a realist synthesis on reducing refusals through improved care interactions during person care interactions between people living with dementia and caregivers. This protocol sets out an important area of research that will likely result in practical recommendations and guidance for professional and informal carers that can improve the quality of care and relationships for those who need support with personal care activities.

The manuscript is well written. Below are minor comments for the authors to consider:
RESPONSE: Many thanks for your comments and clear suggestions for revision.

Abstract, Introduction, Line 46: "and for whom" could be deleted as this is already mentioned by referring to caregivers and people with dementia.
RESPONSE: This has been deleted.

Abstract, Methods and analysis, page 3 line 9: "match informing evidence with" could be "organise related evidence to"
RESPONSE: Many thanks, this has been amended.

Strengths and limitations of this study, bullet point 4, page 3 line 50-52: Suggest this bullet point is strengthened to demonstrate the positive reason for prioritising features rather than focusing on time constraints. Realist review does not seek to give an exhaustive account of what is occurring but instead attempts to explain the relationships of the key factors. I suggest the authors instead indicate what they are likely to base their decision on.
RESPONSE: Many thanks for your comment, we were guided by the editor to amend this section to

highlight only key strengths and limitations. We have amended the 4th bullet point to:
“The project team may have to prioritise key features of care interactions or settings based on relevance, plausibility, and preliminary evidence levels.”

Page 5, line 51: as with the abstract, the "and for whom" could be deleted.
RESPONSE: Many thanks, this has been deleted.

Page 6, line 3: "develop a programme theory", I suggest this is changed to "Develop programme theories" as this is inline with realist review methodology and would be consistent with the use of programme theories throughout the rest of the manuscript.
RESPONSE: Many thanks, this has been amended.

Page 7, lines 5 - 11. The first part of this sentence would benefit from rewording.
RESPONSE: Many thanks, we have deleted the first part of this sentence and amended the following part.

Page 7, lines 52 to 58. Not sure I follow this sentence. Is it that analysing this data will be used to inform topic guides for stakeholder interviews or is it that analysis will seek to explain the factors involved in actions for care, reactions to care interactions and potentially helpful strategies for working with refusals of care? It may be that the sentence is too long and breaking it down into smaller components would help the reader.
RESPONSE: Many thanks for highlighting this. The sentence has been amended and now reads:
“The first author will analyse these datasets with a realist lens examining a range of complexities that potentially interact. Analysis will seek to explain the factors involved in actions for care, reactions to care interactions, and potentially helpful strategies for working with refusals of care.”

Page 8, line 5: it might be helpful to clarify what "methodological categories" refers to.
RESPONSE: Many thanks, this term has now been changed to observation or interview derived data.

Page 8, line 10: should "adding" be add?
RESPONSE: Yes, this has been amended.

Page 8, line 52: perhaps the addition of "Existing" before mid-range theories would be helpful. Please also include some commentary on how these theories will be searched for (e.g. will it be from existing knowledge of the research team and targeted searches for theories from the developing programme theories?).
RESPONSE: Many thanks, we have added ‘existing’ and amended the sentence, which now reads:
“Existing mid-range theories will be sought through targeted searches, led both from the existing knowledge of the research team and from the developing programme theories. These will be examined and, if relevant, incorporated to guide our theory building.”

Page 9, lines 3 - 5. I am not sure I understand what the authors mean by "structured and exploratory searches". Perhaps an additional sentence or two here could be used to clarify the meaning.
RESPONSE: In line with the editor’s comments to add a supplementary file of search terms, this sentence has now been changed.

Page 9, line 26: just a suggestion, but I'm wondering if "influence" is a better word than "change" in relation to Kitwood's impact on dementia care.
RESPONSE: Many thanks, we have amended the sentence.

Page 10, lines 21 - 25: The sentence starting "Where concepts..." could be read that the authors are only seeking evidence that aligns with their current understanding and initial programme theories.

Consider rewording as one of the ways realist review addresses challenges of bias is by adjudicating between alternative explanations.
RESPONSE: Many thanks for highlighting this important issue, we have added more information and this part now reads:
“Where concepts such as refusals of care are not portrayed accurately or the meaning authors are ascribing mismatches our conceptualisation (such as those centred on non-adherence or refusal of medical procedures), we will exclude articles. However, articles offering alternative explanations within our conceptualisation will be included and used to challenge biases and team interpretations.”

Page 10 Line 36: refer to IF THEN BECAUSE statements for consistency.
RESPONSE: Many thanks, this has now been amended.

Page 10 lines 35 - 39: "context-mechanism-outcome configurations and testing.." I think the and should be for.
RESPONSE: Many thanks, we have amended the sentence.

Page 10 line 54: "Mapping to" could instead be "Evidence relating to"
RESPONSE: Many thanks, this has been amended.

Page 11 line 12: "redeveloping" could instead be "reworking" or "revising" - redeveloping, to me, suggests starting over rather that using the data to further shape and strengthen initial ideas.
RESPONSE: Many thanks for highlighting this, which has been amended to revising.

Page 11, line 21: "One" is missing before "Researcher", "the" is missing before "theoretical underpinnings", "of programme considering" one or several words missing here.
RESPONSE: Many thanks, this sentence has been amended.

Page 11, Line 25 - 27: The sentence "Consideration will be given to
formal and informal/unintended consequence causal insights." needs rewording.
RESPONSE: Many thanks, this sentence has been reworded:
“Consideration will be given to both intended and unintended causal insights.”

Page 11, line 27: "Prioritised initial programme theories" there needs to be an earlier discussion of how initial programme theories will be prioritised, what will be the justification for how theories are prioritised.
RESPONSE: Many thanks for highlighting, a new sentence reads:
“From these initial programme theories, the team will agree on the scope and focus of the realist synthesis, if necessary, prioritising initial programme theories to take forward based on their relevance, plausibility, and preliminary evidence levels.”

Page 11 line 46: the authors state that recommendations and conclusions will be tested and refined in stakeholder interviews - does this mean that working recommendations and conclusions will be developed before interviews with stakeholders? To me, this seems like a separate, later piece of work. I would anticipate interviews would explore stakeholder reactions to the programme theories and enhance the validity but as insights continue to be gathered perhaps agreeing recommendations and conclusions following integrating evidence from these interviews is an additional activity?
RESPONSE: Many thanks for your comment, we have tempered our aims for the stakeholder interviews with recommendations and conclusions developing post-interviews after evidence synthesis.
“We will use stakeholder interviews to explore reactions to the programme theories and enhance validity. … Key insights and nuances arising during interviews will be integrated with existing evidence to refine programme theories from which, recommendations and conclusions will be developed.”

Page 13, line 13 "Then" should be "The".
RESPONSE: Many thanks, this has been amended.

Suggestion for throughout the manuscript: to change "Evidenced programme theories" to "Evidence-based programme theories". While the theories will draw on a significant amount of evidence they will only ever remain theories.

RESPONSE: Many thanks, this has been amended throughout.
